# Distinct trajectories of subjective cognitive decline before diagnosis of neurocognitive disorders: Longitudinal modelling over 18 years

**DOI:** 10.1002/alz70857_096805

**Published:** 2025-12-24

**Authors:** Tau Ming Liew

**Affiliations:** ^1^ Singapore General Hospital, Singapore, Singapore; Saw Swee Hock School of Public Health, National University of Singapore, Singapore, Singapore; Duke‐NUS Medical School, Singapore, Singapore

## Abstract

**Background:**

Subjective cognitive decline (SCD) is an established predictor of neurocognitive disorders (NCD) (i.e. mild cognitive impairment and dementia). Yet, its construct remains contentious. Many individuals with SCD do not progress to NCD, leading to an alternative term in the literature – ‘functional cognitive disorders’ – to describe the SCD experience in these individuals. This study examined the distinct differences in trajectories of subjective cognitive decline (SCD), between those who did and did not eventually develop NCD.

**Method:**

This case‐control study included 5,167 participants aged ≥50 years, followed‐up near‐annually to evaluate for SCD and NCD (median follow‐up=8.1 years; range=1.0–18.0). *Cases* were defined as those who developed incident NCD during follow‐up; *controls* completed ≥10 years of follow‐up and had normal cognition throughout follow‐up period. SCD trajectories were modelled with mixed‐effect logistic regression, using a backward timescale.

**Result:**

Those who developed NCD (*cases*) had new onset of SCD within past 20 years, which became particularly noticeable 13–14 years before diagnosis, and became even more prominent in the last 4 years. Those who did not develop NCD (*controls*) reported SCD since younger age, with the probability of SCD remaining constant over time. The distinctive trajectories were consistent across Alzheimer's & non‐Alzheimer's disease, and among those with higher baseline rates of SCD due to psychiatric conditions.

**Conclusion:**

SCD has distinctive trajectories among those who do and do not progress to NCD. The distinctive trajectories can inform NCD risk for early interventions, and guide public health messaging to distinguish high‐risk SCD from normal ageing. Future SCD scales may possibly need to evaluate for symptom changes over a longer, 20‐year horizon to better capture new onset of SCD within this longer timeframe.

